# Supplementary material for: Splice-Junction-Based Mapping of Alternative Isoforms in the Human Proteome
Source: Cell Rep. Author manuscript; Available in PMC 2020 Jan 15. (PMC6961840; doi:10.1016/j.celrep.2019.11.026)
Supplement: 2 [file NIHMS1546469-supplement-2.zip › DF1/PXD010154/Testis/Testis_23_SRSF11_VTAQPDVLEVQAEYITAGPGPSGGPGGGG.pdf]

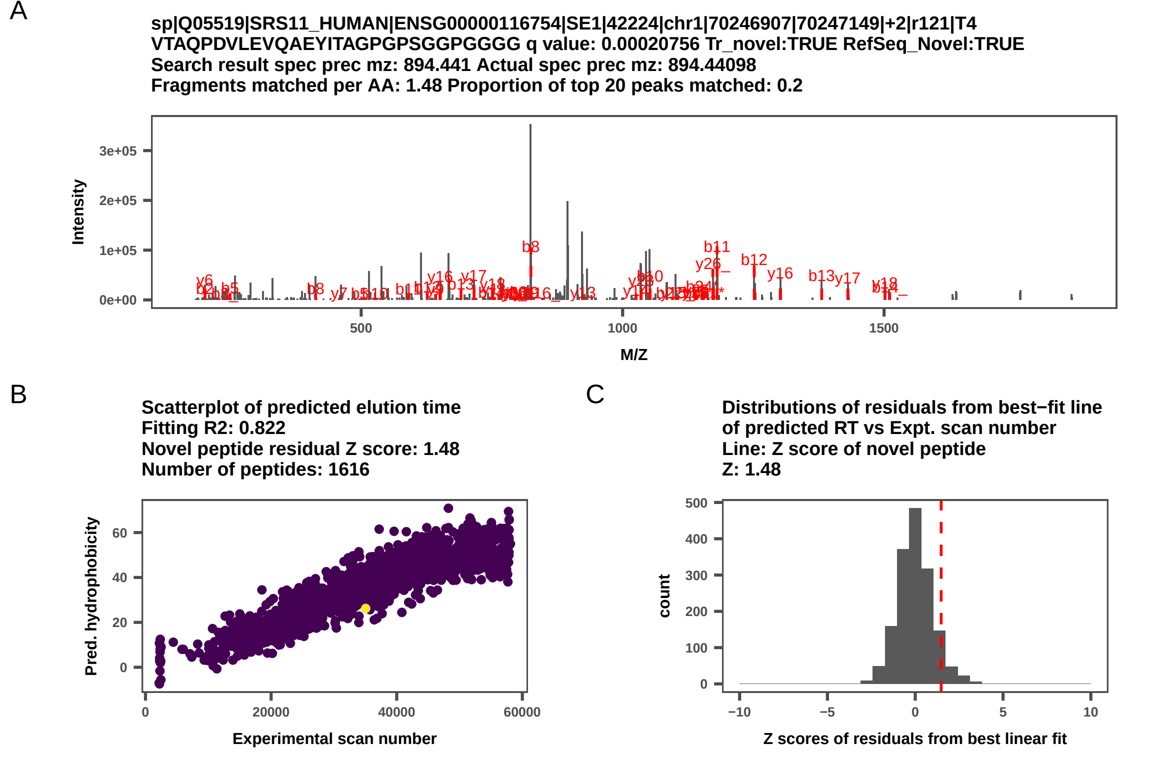
A
sp|Q05519|SRS11_HUMAN|ENSG00000116754|SE1|42224|chr1|70246907|70247149|+2|r121|T4
VTAQPDVLEVQAEYITAGPGPSGGPGGGG q value: 0.00020756 Tr_novel:TRUE RefSeq_Novel:TRUE
Search result spec prec mz: 894.441 Actual spec prec mz: 894.44098
Fragments matched per AA: 1.48 Proportion of top 20 peaks matched: 0.2
Intensity
3e+05
2e+05
1e+05
0e+00
M/Z
500
1000
1500
b8
b11
b12
y16
b13
y17
y13
B
Scatterplot of predicted elution time
Fitting R2: 0.822
Novel peptide residual Z score: 1.48
Number of peptides: 1616
Pred. hydrophobicity
Experimental scan number
0
20
40
60
20000
40000
60000
C
Distributions of residuals from best−fit line
of predicted RT vs Expt. scan number
Line: Z score of novel peptide
Z: 1.48
count
500
400
300
200
100
0
−10
−5
5
10
Z scores of residuals from best linear fit
